# How Bristol Royal Infirmary Has Watched the World Change
*By courtesy of the *Editor of the Bristol Evening Post*.


**Published:** 1937

**Authors:** E. Watson-Williams

**Affiliations:** Assistant Editor


### HOW BRISTOL ROYAL INFIRMARY HAS WATCHED THE WORLD CHANGE.*

BY

E. Watson-Williams, M.C., F.R.C.S.,
*Assistant Editor.*

The date 20th June has for Bristolians a double significance, for on 20th June, 1837, Queen Victoria came to the throne, and exactly one hundred years earlier (on 20th June, 1737) the Bristol Royal Infirmary first opened for the reception of patients, though the "official" opening was deferred until 13th December.

During the whole of these two hundred years the work of the Infirmary has continued by day and by night, unceasing and increasing. But it is difficult for us now fully to appreciate what this means—how really different was the Great Britain which the Infirmary's founders knew from that of to-day.

How many, in fact, realize that the Infirmary was opened in the reign of George II, and in the year of the death of his Queen, Caroline of Anspach ? It was already well settled to work when the Battles of Dettingen and of Fontenoy were fought; when Charles Edward Stuart invaded England; when Frederick the Great came to his throne; when the first beginnings of the English novel appeared with Richardson's *Pamela*, Dr. Smollett's *Roderick Random*, and Fielding's *Tom Jones ;* when Swift was writing to Pope, and the strange new sect of Methodists appeared in London.

* By courtesy of the *Editor of the Bristol Evening Post.*

In those days the British Empire, in which Ireland was a separate kingdom, consisted of a few small islands, Gibraltar, and the eastern coast of North America. Canada was French, South Africa Dutch, Ceylon Portuguese, India independent (except where the French had established themselves on the coast), and Australia unexplored. There were no railways, of course (the steam engine had not been invented), there were no newspapers, and hardly any roads that we should recognize as such, there were no pavements at all !

The calendar had not been "reformed," so that the year ended in March instead of December (hence the mistake in the date carved over the Infirmary door subsequently) ; surgeons were still (some at least) "barber-surgeons," while a licence to practise medicine could be obtained from a bishop ; a pound of bread, a pound of beef, and four pints of beer was the regular daily ration for Infirmary patients.

The Infirmary was twenty years old when Mozart was born, and the Black Hole of Calcutta was followed by Clive's victory at Plassey, and British rule in India began. Soon after came the Battle of Minden, Wolfe's capture of Quebec, which gave Canada to Britain, and the accession of George III.

The Infirmary was thirty years old when the quarrel with the American Colonies began, and the steam engine and the "spinning jenny" were invented ; Captain Cook made his first voyage to Australia (and showed the importance of what we now call vitamins !) ;* soon after Parliamentary debates were allowed to be published ; newspapers (*e.g. The Times*) and the Royal

* For although since the time of Sir James Lancaster (*circa* 1592) East Indiamen were supplied with oranges and lemons for the prevention of scurvy, the Royal Navy ignored this knowledge until about 1790.—EDITOR.

Academy were founded; and Dr. Johnson set out on his tour of the Hebrides.

When the Infirmary was already fifty years old came the trial of Warren Hastings and the French Revolution; and when it was sixty years old Jenner proved the value of vaccination, and John Harvey's firm, of sherry fame, was founded.

A little later, when the upstart Napoleon Buonaparte became "First Consul" of the French, Humphry Davy at the Bristol Pneumatic Institute discovered the anæsthetic powers of "laughing gas"; Ireland was united with Great Britain, Malta was captured, and Ceylon and South Africa were added to Great Britain.

The Infirmary was seventy years old when the slave trade was abolished,* soon after the Battle of Trafalgar, and nearly eighty years old when the Bristol Eye Hospital and Eye Dispensary were founded, when Laënnec invented the stethoscope, the Battle of Waterloo was fought, and Napoleon sent to St. Helena.

George IV reigned and William IV came to the throne; the Infirmary was over ninety years old, and the first railway was opened between Liverpool and Manchester; the Bristol riots marked the first Parliamentary Reform Act; the Bristol General Hospital and Bristol Medical School were founded.

Quite at the end of the Infirmary's century came the first anniversary meeting (at the Infirmary) of the Provincial Medical Association, now the B.M.A.; Reform of the Poor Law; the beginnings of national

* The evidence given by an Infirmary student, Falconbridge. before the Parliamentary Committee is said to have been the chief factor that induced the Committee to report in favour of abolition. Falconbridge had the rare experience of surviving four voyages to West Africa as an "African (*i.e.* Slave-ship) Surgeon."—Editor.

education ; the general Registration Act ; the *Pickwick Papers ;* and the accession of Victoria.

Even then, after a hundred years' work, the Infirmary looked out on a world very different from this. Salmon were still caught in the Thames ; lighting by gas was a startling experiment ; iron ships were unknown ; duelling was frequent ; "bleeding" was a commonplace measure ; anæsthetics had not come into use ; beer was a usual drink for children of six ; there were no postage stamps and (think of it !) no Income Tax.

The triumphs of the succeeding hundred years we need not recapitulate. In the medical field they include the discovery of anæsthesia, the demonstration (by Dr. Budd of the Royal Infirmary) that typhoid and phthisis are infectious—the real starting-point of modern sanitation and public health—and the revolution in surgery brought about by Lister's antiseptic methods.

At least it cannot be denied that the preceding century saw Britain rise from mediocrity to the greatest Empire and the strongest Power in the world ; while internally the abolition of slavery, of religious disqualifications, and of censorship of the Press, the introduction of Parliamentary Reform, and the development of steam power wrought fundamental changes in the lives and the thoughts of our countrymen.

And when we recall that all this took place during the first hundred years of the Infirmary's work, we can then begin to understand how truly venerable is the oldest provincial teaching hospital in the kingdom.

